# Supplementary material for: Preclinical Enzyme Replacement Therapy with a Recombinant β-Galactosidase-Lectin Fusion for CNS Delivery and Treatment of GM1-Gangliosidosis
Source: Cells. 2022 Aug 19;11(16):2579. doi: 10.3390/cells11162579 (PMC9406850; doi:10.3390/cells11162579)
Supplement: Supplementary file 1 [file cells-11-02579-s001.zip › cells-1831160-supplementary.pdf]

Supplementary Materials

Preclinical Enzyme Replacement Therapy with a Recombinant β-Galactosidase-Lectin Fusion for CNS Delivery and Treatment of GM1-Gangliosidosis

Jason A. Weesner[1,2], Ida Annunziata[1,3], Tianhong Yang[4], Walter Acosta[4], Elida Gomero[1], Huimin Hu[1], Diantha van de Vlekkert[1], Jorge Ayala[4], Xiaohui Qiu[1], David N. Radin[4], Carole L. Cramer[4*] and Alessandra d'Azzo[1,2*]

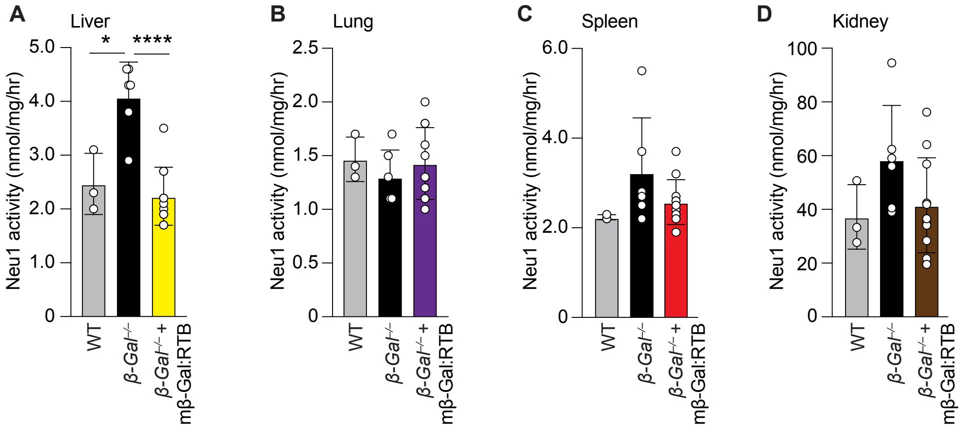

Figure S1. Neuraminidase activity in mβ-Gal:RTB treated mice. (**A–D)** Neu1 activity measured in the liver (A), lung (B), spleen (C), and kidney (D) from WT, *β-Gal*$^{-/-}$ and treated mice showed a significant increase in the *β-Gal*$^{-/-}$ liver and is normalized to WT levels following treatment. Both spleen and kidney followed a similar trend as the liver, whereas the lung showed no difference. WT: $n = 3$, *β-Gal*$^{-/-}$: $n = 6$, mβ-Gal:RTB: $n = 11$. Data represents the means ± SD, $^{*}p < 0.05$, $^{****}p < 0.0001$.

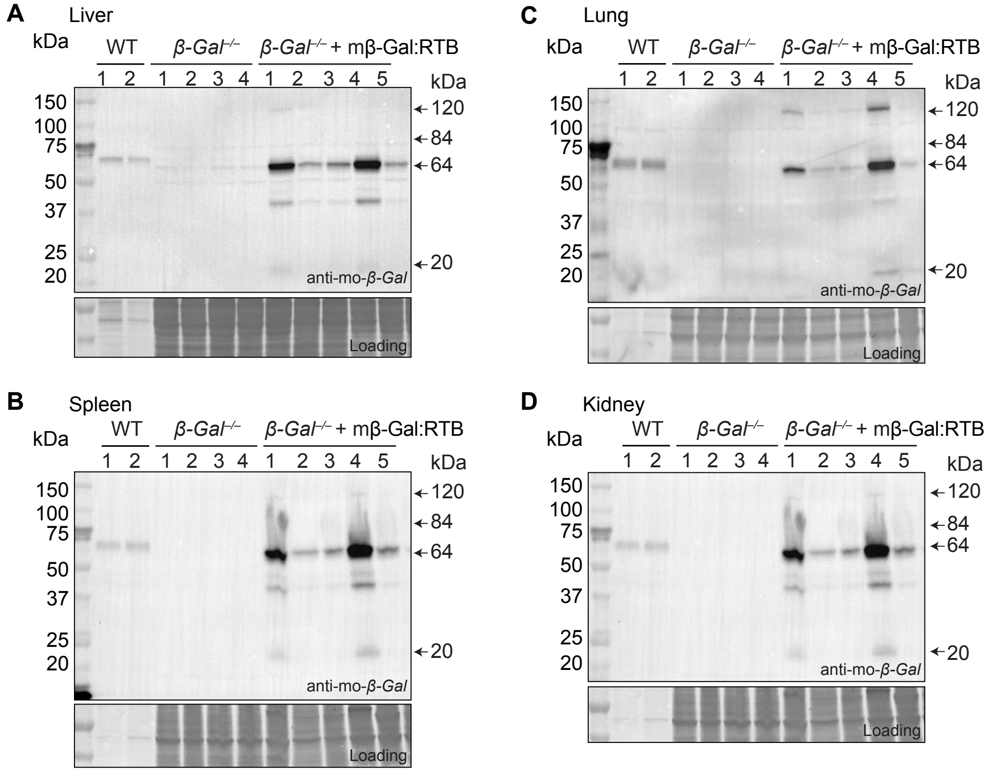

Figure S2. Long term treatment with mβ-Gal:RTB in visceral organs. (**A–D**) Second set of Immunoblot analyses of liver (A), lung (B), spleen (C) and kidney (D) used for the protein quantification in Main Article Figure 3I–L.

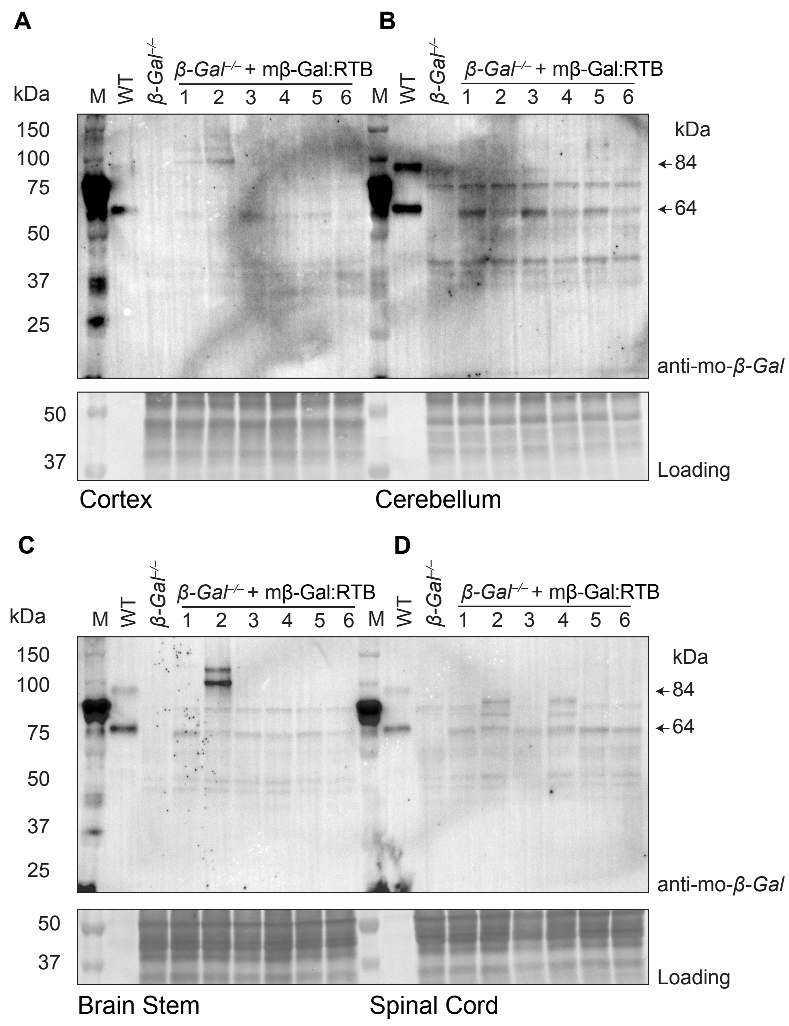

Figure S3. Long term treatment with mβ-Gal:RTB in CNS. (**A–D**) Second set of Immunoblot analyses of cortex (A), cerebellum (B), brain stem (C) and spinal cord (D) used for the protein quantification in Main Article Figure 5F, H, J, and L.

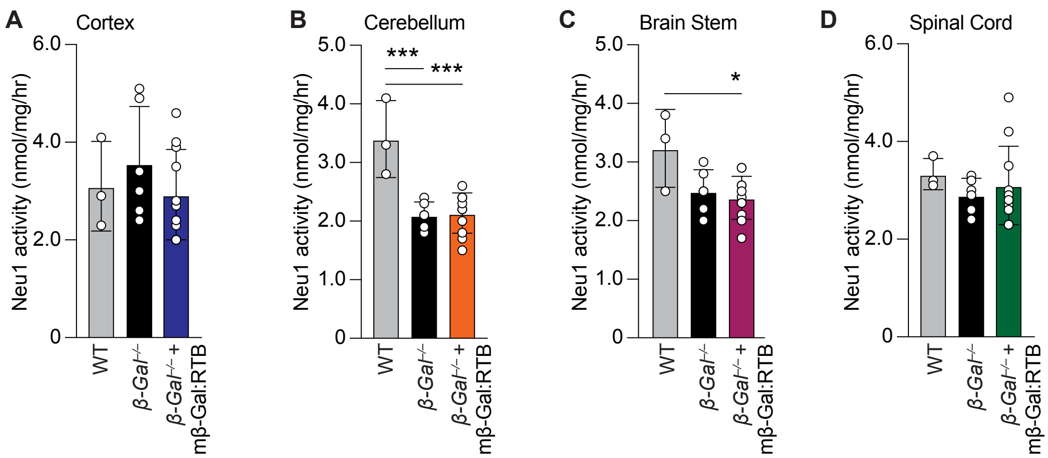

Figure S4. Neuraminidase activity in the CNS of mβ-Gal:RTB treated mice. (**A–D)** Neu1 activity measured in the cortex (A), cerebellum (B), brain stem (C), and spinal cord (D) from WT, *β-Gal*$^{-/-}$ and treated mice. WT: $n = 3$, *β-Gal*$^{-/-}$: $n = 6$, mβ-Gal:RTB: $n = 11$. Data represents the means ± SD, *$p < 0.05$, ***$p < 0.001$.

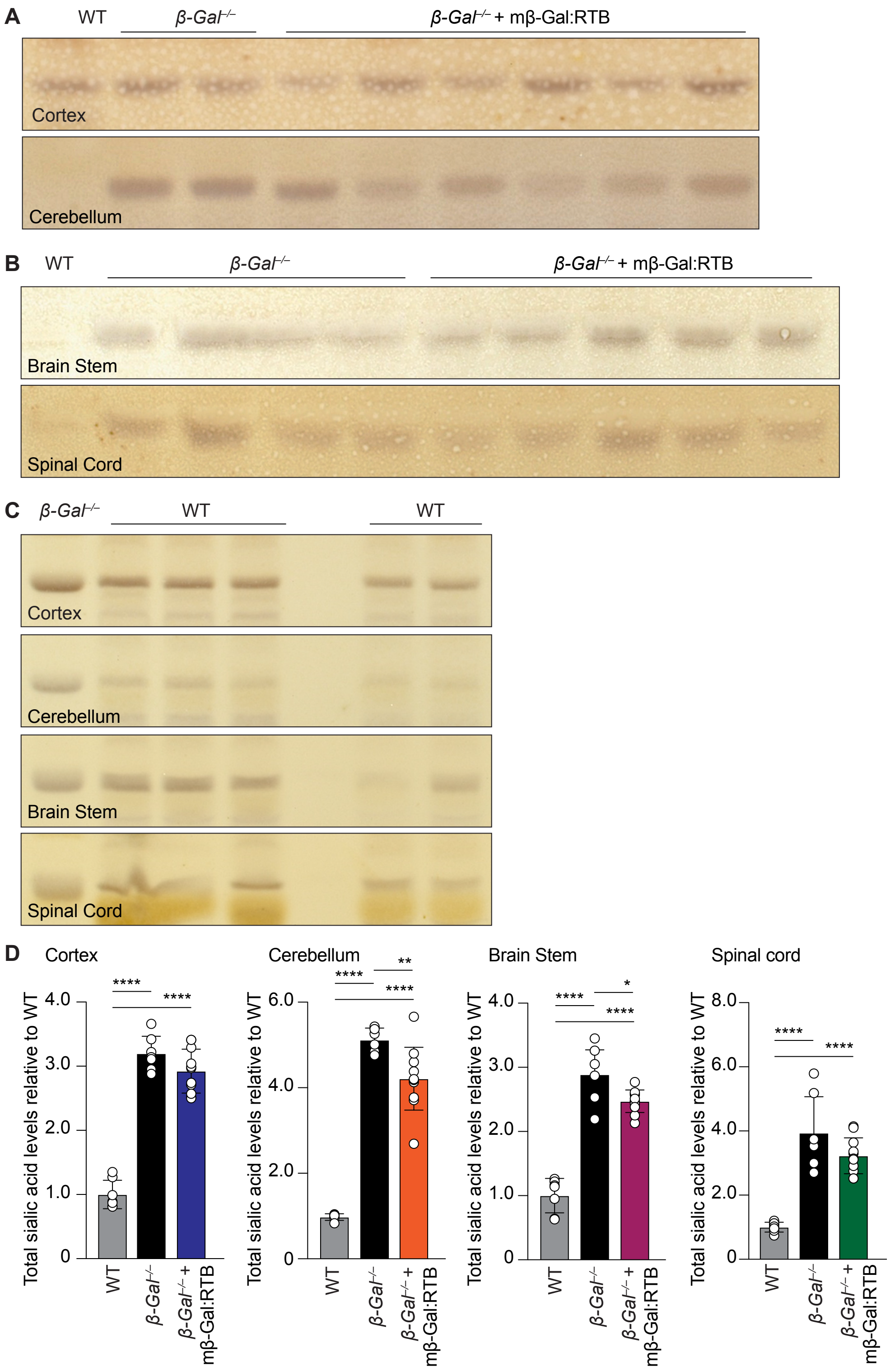

Figure S5. mβ-Gal:RTB treated mice show reduction of GM1 in the CNS. (**A-C**) HPTLC analyses of GM1 levels in the CNS regions of WT, *β-Gal*$^{-/-}$ and treated mice. (**D**) Quantification of total bound sialic acid measured in WT, *β-Gal*$^{-/-}$ and treated mice showed increased levels in the *β-Gal*$^{-/-}$ mice which was partially reduced in the cortex and spinal cord and significantly reduced in the cerebellum and brain stem in treated mice compared to the *β-Gal*$^{-/-}$. WT: $n = 7$, *β-Gal*$^{-/-}$: $n = 7$, mβ-Gal:RTB: $n = 11$. Data represents the means ± SD, *$p < 0.05$, **$p < 0.01$, ****$p < 0.0001$.

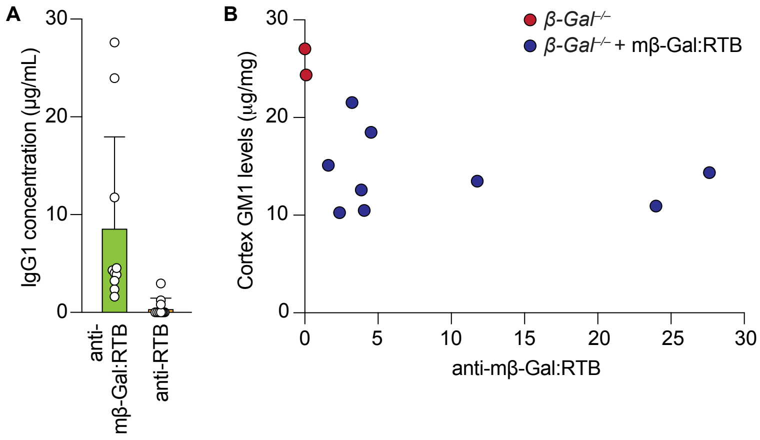

Figure S6. Correlation analysis between anti-drug IgG1 levels and therapeutic response. (**A**) Serum antibody response against mβ-Gal:RTB and RTB proteins. n = 11 (**B**) Correlation analysis between anti-drug IgG1 levels in sera and GM1 levels in the cortex of treated mice. *β-Gal*$^{-/-}$: n = 2, mβ-Gal:RTB: n = 9.

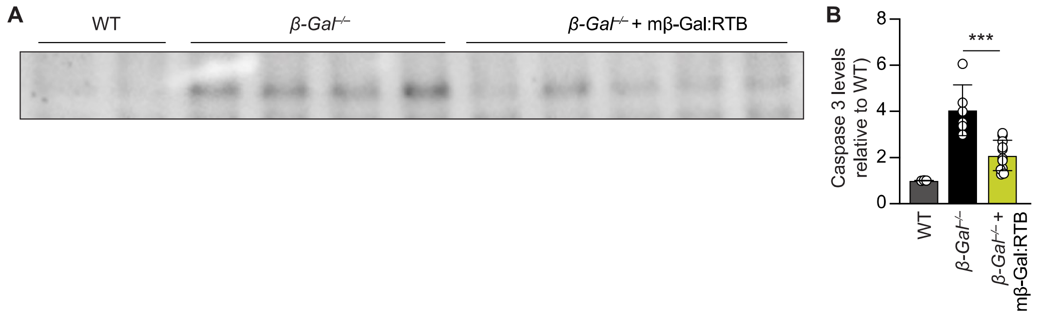

Figure S7. mβ-Gal:RTB treated mice have reduced caspase-3 protein levels. (**A**) Immunoblot analysis of casepase-3 levels in WT, *β-Gal*$^{-/-}$ and mβ-Gal:RTB injected mice using anti-caspase-3 antibody. (**B**) Quantification of caspase-3 levels in A. WT: $n = 3$, *β-Gal*$^{-/-}$: $n = 6$, mβ-Gal:RTB: $n = 11$. Data represents the means ± SD, $^{***}p < 0.001$.

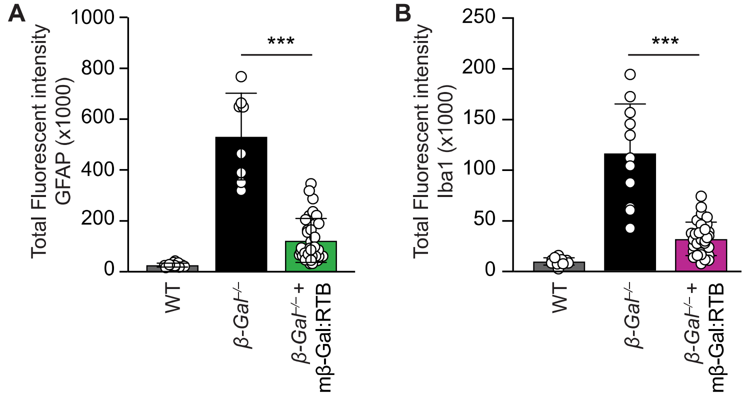

Figure S8. 24h dose-dependent treatment of mβ-Gal:RTB in *β-Gal*$^{-/-}$ mice. (**A**) Quantification of IF staining of GFAP WT: $n = 12$, *β-Gal*$^{-/-}$: $n = 8$, mβ-Gal:RTB: $n = 33$ from figure 8. (**B**) Quantification of IF staining of IBA1 WT: $n = 11$, *β-Gal*$^{-/-}$: $n = 11$, mβ-Gal:RTB$n = 31$ from figure 9. Data represents the means ± SD, ***$p < 0.001$.

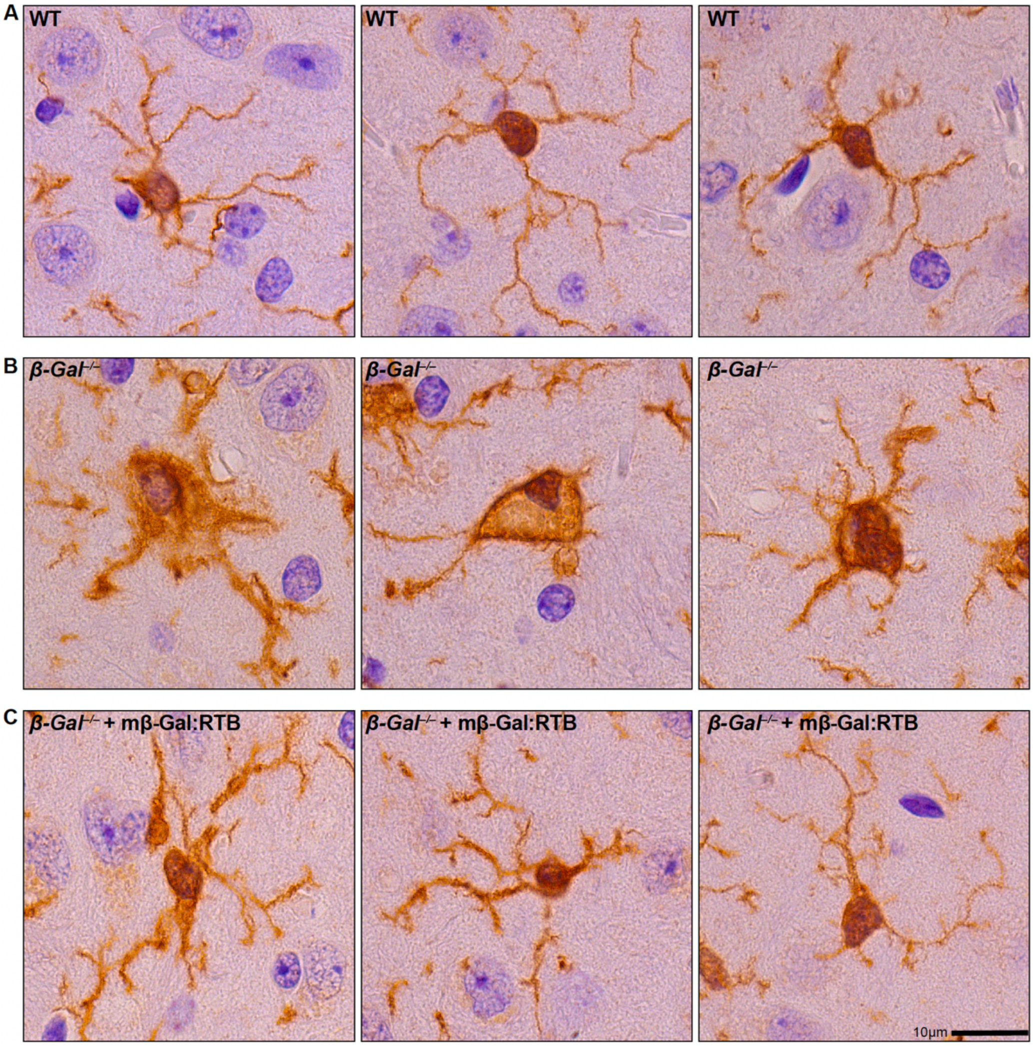

Figure S9. Microglia size and shape show less microgliosis in thalamus of treated mice. (**A** and **B**) IHC using anti-IBA1 antibody show (A) ramified WT microglia and (B) amoeboid microglia in *β-Gal*$^{-/-}$ with varying degrees of severity. (**C**) representative images of mβ-Gal:RTB treated microglia shows the range of amoeboid-like (left) to ramified (right) microglia that exist following treatment. Scale bar: 10 μm.
